# Enhancing Quality of Life in Head and Neck Cancer: A Scoping Review on the Role of Physical Prehabilitation

**DOI:** 10.1002/cam4.71743

**Published:** 2026-03-27

**Authors:** Alice Maria Santagostino, Mauro Parozzi, Giovanni Cangelosi, Sara Morales Palomares, Stefano Mancin, Giulia De Pasquale, Fabio Petrelli, Marco Sguanci, Beatrice Mazzoleni

**Affiliations:** ^1^ IRCCS Humanitas Research Hospital Milan Italy; ^2^ School of Nursing, “San Paolo” Campus, Asst Santi Paolo e Carlo University of Milan Milan Italy; ^3^ Units of Diabetology ASUR Marche Marche Italy; ^4^ Department of Pharmacy, Health and Nutritional Sciences (DFSSN) University of Calabria Rende Italy; ^5^ School of Pharmacy Polo Medicina Sperimentale e Sanità Pubblica “Stefania Scuri” Marche Italy; ^6^ A.O. Polyclinic San Martino Hospital Genova Italy; ^7^ Department of Biomedical Sciences Humanitas University Milan Italy

**Keywords:** chemotherapy, head and neck cancer, prehabilitation, quality of life, radiotherapy

## Abstract

**Background/Aim:**

Head and neck cancer presents a significant challenge for patients. Beyond the direct impact of the disease, chemotherapy and radiotherapy, while essential for cancer control, can further compromise patients' quality of life (QoL) and emotional well‐being, while also introducing additional complications. These treatments may exacerbate existing cancer‐related symptoms and contribute to malnutrition. This review aimed to identify and evaluate physical prehabilitation interventions implemented before or during radiotherapy/chemotherapy treatments to determine their impact on the QoL of patients with head and neck cancer.

**Methods:**

This scoping review was structured according to the framework proposed by the Joanna Briggs Institute (JBI) and reported following the Preferred Reporting Items for Scoping Reviews (PRISMA‐ScR) guidelines.

**Results:**

Eight studies were included, encompassing a total sample of 819 subjects (397 in experimental groups). The interventions identified included exercises and stretching to prevent mobility issues and trismus, home‐based training programs with periodic supervision, comprehensive programs with supervised physiotherapy sessions, jaw mobility exercises, dysphagia therapy, oral exercises, and preventive rehabilitation. A positive association was found between prehabilitation interventions and improved QoL in patients. Customizing interventions based on patient characteristics and treatment modalities was shown to enhance the effectiveness of these programs.

**Conclusions:**

Prehabilitation interventions represent a holistic and functional approach to improving patient outcomes and QoL. Further research is needed to refine these approaches and optimize the overall QoL of head and neck cancer survivors.

## 1 Introduction

Head and neck cancer pose a significant challenge for both patients and healthcare professionals, impacting not only physical health but also psychological well‐being [1]. Beyond the immediate effects of the disease, treatments such as chemotherapy and radiotherapy, while crucial for cancer management, can further compromise patients' quality of life (QoL) [2]. QoL is a multifaceted concept encompassing the physical, psychological, social, and functional dimensions of an individual's well‐being [3]. In patients with head and neck cancer, these dimensions are often severely affected due to various factors. For example, physical symptoms such as pain, dysphagia, dysarthria, and dysfunction of the jaw and mouth can significantly impair both the physical and functional components of QoL [4]. Furthermore, the emotional burden of the disease is substantial, with many patients experiencing anxiety, depression, and body image issues, which not only intensify psychological distress but also negatively influence social interactions and physical health [5].

In addition to the inherent challenges of the disease, the treatments themselves introduce further complications that can exacerbate QoL issues [6]. Chemotherapy, while essential, often leads to side effects such as nausea, fatigue, loss of appetite, dysgeusia, xerostomia, and oral mucositis, all of which can significantly limit daily activities and diminish self‐esteem [7, 8, 9, 10]. Similarly, radiotherapy adds to the burden by causing skin burns, inflammation, and worsening swallowing difficulties, intensifying cancer‐related symptoms. These side effects collectively contribute to a decline in overall QoL and frequently result in malnutrition, characterized by progressive weight loss and deficiencies in macro‐ and micronutrients [11, 12, 13]. In response to these challenges, there has been increasing interest in prehabilitation, a proactive approach aimed at optimizing patients' physical, emotional, and social health before the initiation of cancer treatments [14]. Prehabilitation is a multidisciplinary intervention designed to prepare patients for the physical demands of oncological treatments by enhancing their overall health and functional capacity, thereby improving treatment tolerance and minimizing side effects [15, 16]. This approach involves a coordinated effort from a team of healthcare professionals—including physicians, nurses, physiotherapists, speech therapists, dietitians, and psychologists—who tailor personalized interventions to meet the specific needs of each patient [17, 18]. Central to prehabilitation is the goal of improving muscle strength, endurance, flexibility, and overall physical function through targeted exercise programs, while also enhancing nutrition, pain management, and mental well‐being.

Emerging evidence suggests that prehabilitation provides significant benefits for patients with head and neck cancer, helping to mitigate declines in physical function and improve QoL throughout the treatment process [19, 20]. As research in this field progresses, clinical trials are exploring the efficacy of various prehabilitation strategies and identifying patient populations that stand to benefit the most from these interventions [21, 22]. Ultimately, the aim is to integrate prehabilitation as a standard component of the care pathway for patients with head and neck cancer, thereby enhancing their QoL and overall well‐being during the course of their treatment.

### 1.1 Objective

The objective of this scoping review is to identify and evaluate physical prehabilitation interventions performed before or during radio/chemotherapy treatments, in order to determine their impact on the QoL of patients with head and neck cancer.

## 2 Methods

### 2.1 Study Design

This review follows the Joanna Briggs Institute (JBI) scoping review methodology [23] and is reported in accordance with the guidelines outlined in the Preferred Reporting Items for Scoping Reviews (PRISMA ScR) framework [24]. Additionally, building on insights from a previous study [25], which called for more transparency and detail in the procedural stages, as well as the incorporation of quality assessment into the scoping review approach, we have adopted these recommendations [26, 27]. In this review, we integrate a critical appraisal of the quality of the selected studies, using the JBI methodology to assess the rigor of the included research. The scoping review protocol was prospectively registered on the Open Science Framework, available at: https://doi.org/10.17605/OSF.IO/9JKHP.

### 2.2 Identification of the Research Question

The research question for this study was developed using the PCC framework [26], which is commonly used in scoping reviews to guide topic formulation. The PCC framework focuses on three core elements: Population (P), Concept (C), and Context (C). For this review, the following elements were considered: P: patients with head and neck cancer; C: identification of primary and secondary research studies that describe prehabilitation interventions aimed at improving various aspects of patient QoL; C: Hospital and community setting.

### 2.3 Eligibility Criteria

The inclusion criteria encompassed any type of primary study, including experimental, observational, qualitative, and mixed‐method designs, with a focus on physical prehabilitation interventions aimed at improving QoL. These interventions had to be initiated and performed either before or during the initiation of chemotherapy and/or radiotherapy. The participants were required to be individuals over the age of 18, newly diagnosed with head and neck cancer, and scheduled to undergo anticancer treatments. The exclusion criteria included studies that did not involve head and neck cancer, as well as those not available in full text, such as books, chapters, study protocols, or congress contributions.

### 2.4 Search Strategy

The search was conducted in June 2024 in the databases PubMed/Medline, Embase, CINAHL, and Cochrane Library using the keywords: “radiotherapy,” “chemotherapy,” “head and neck cancer,” “quality of life,” “prehabilitation,” and their variations, appropriately combined by Boolean operators. Additionally, a manual search was performed by scanning the reference lists of relevant articles and Google Scholar to retrieve additional records from gray literature. Full search algorithms are available in the data availability statement (Table S1).

### 2.5 Selection of Evidence Sources

The selection of evidence sources was conducted according to the rigorous guidelines set by the JBI methodology framework, ensuring both a systematic and thorough approach. Two independent researchers (AMS and GDP) were responsible for selecting and including publications in the review. Each researcher independently evaluated all titles and abstracts retrieved from electronic database searches. Duplicates and irrelevant records were systematically removed using EndNote 20 software (https://endnote.com/). In cases of discrepancies, a third reviewer (SM) was consulted to resolve any conflicts. After the initial screening, the full texts of the remaining studies were retrieved, and two reviewers (AMS and GDP) independently assessed their eligibility based on predefined inclusion criteria. Discrepancies were resolved through consensus meetings, with a third reviewer (SM) acting as arbitrator.

### 2.6 Evaluation of Risk of Bias and Methodological Quality of Studies

The risk of bias and the methodological quality of the included studies were independently evaluated by two researchers (xx and xx) using the JBI Critical appraisal tools. Any disagreements were resolved by an impartial third‐party reviewer (xx). Study quality was determined based on criteria established in a previous study [28]. In this analysis, studies with a JBI score greater than 70% were considered of high quality, those scoring between 50% and 70% were considered of medium quality, and those scoring below 50% were classified as low quality. The complete algorithm used to assess study quality can be found in Tables S2–S5.

### 2.7 Data Extraction and Synthesis

The following data were extracted: authors, publication date, study design, country, study sample, prehabilitation interventions, QoL assessment method, and results of the included studies. The results of this scoping review were categorized based on the identified prehabilitation interventions, with their effectiveness in improving QoL summarized using narrative synthesis, supported by tables and figures.

## 3 Results

**FIGURE 1:**
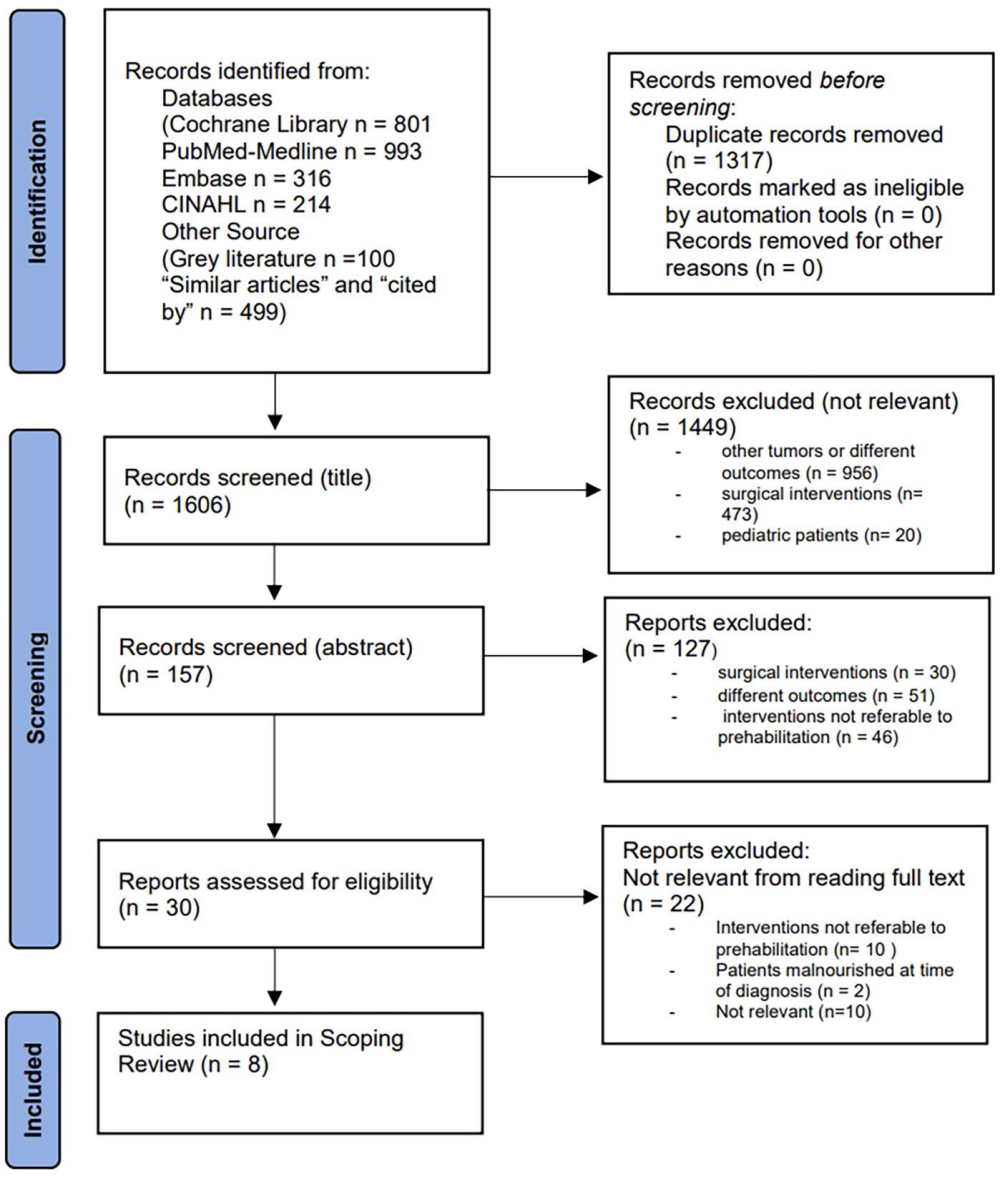
PRISMA ScR flowchart.

A total of 2923 articles were identified through database searches: 801 from the Cochrane Library, 993 from PubMed/Medline, 316 from Embase, 214 from CINAHL, and 599 from other sources. After removing 1317 duplicates, all titles were screened, and 157 articles were retained for abstract review to evaluate their eligibility. Of these, 127 were deemed irrelevant, and the remaining 30 full‐text articles were assessed; 22 of these were subsequently excluded as they did not meet the selection criteria for our research. The screening process ultimately included eight studies in this review (Figure 1).

### 3.1 General Characteristics of the Studies

The studies were conducted primarily in European countries, including Italy, Ireland, Sweden, Denmark, and the Netherlands. The articles featured a variety of study designs, such as combined retrospective analyses and case–control studies [29], randomized controlled trials (RCTs) [30, 31, 32, 33, 34], prospective intervention studies [35], and longitudinal prospective cohort studies [36]. The total patient sample across these studies comprised 819 subjects (397 in experimental groups and 422 in control groups), with individual study sizes ranging from 12 to 374 patients (Table 1). All the included studies demonstrated high methodological quality and a low risk of bias, with studies having a mean JBI score exceeding 70% considered to be of high quality (Tables S2–S5).

**TABLE 1:** Characteristics of the studies included.

Author, year of publication, country	Study design	Sample [*n*]	Principal prehabilitation intervention	Method of assessing QoL	Results	Quality
Carmignani et al. [29], 2018, Italy	Retrospective observational study Two‐arm case–control study	[*n* = 60] [*n* = 12] IG = 6 CG = 6	Tongue resistance exercises Effortful swallow Masako maneuver Mendelsohn maneuver Shaker maneuver	EORTC QLQ‐C30 EORTC QLQ‐H&N35	Patients with hypopharyngeal/laryngeal cancers had better swallowing function and QoL (*p* < 0.05) Prophylactic swallowing exercises improved post‐treatment MDADI (*p* = 0.006) and DHI (*p* = 0.002), enhancing QoL	+++/Low
Pisano Messing et al. [30], 2016, USA	RCT	[*n* = 60] IG = 30 CG = 30	Oro‐motor strength/stretch exercises and swallow maneuvers TheraBite exercise Compensatory swallow strategies and diet modifications	EORTC QLQ‐C30 EORTC H&N35	No significant differences between groups except for QoL global health (difference in score, 10.1, 95% CI, −0.1–20.3; *p* = 0.05)	+++/Low
Mortensen et al. [31], 2015, Denmark	RCT	[*n* = 44] IG = 22 CG = 22	Swallowing exercises (range of motion drills and resistance exercises)	EORTC QLQ‐C30 EORTC QLQ‐H&N35	No difference between groups. EORTC Global health: 83 vs. 79 (*p* = 0.48)	+++/Low
van der Molen et al. [32], 2014, Netherlands	RCT	[*n* = 44] IG = 15 CG = 14	Masako maneuver, super‐supraglottic swallow and use of specific device for passive and slow mouth opening	QoL questionnaire (not validate)^a^	Many initial issues significantly decreased in the first year post‐treatment, except xerostomia (59%)	+++/Low
Høgdal et al. [33], 2014, Denmark	RCT	[*n* = 100] IG = 50 CG = 50	Daily slow dynamic exercises Stretching exercises Self‐administered lymph drainage	EORTC QLQ‐C30 EORTC H&N35	The intervention did not improve health‐related quality of life	+++/Low
Pauli et al. [35], 2014, Sweden	Prospective intervention study	[*n* = 100] IG = 50 CG = 50	Warm up movements (jaw opening and small sideway movements of the jaws) Passive stretching with the jaw mobilizing specific device Five repetitions of active exercise (bite toward resistance)	EORTC QLQ‐C30 EORTC H&N35	IG increase EORTC QLQ‐C30 Global Health (*p* < 0.001)	+++/Low
Ahlberg et al. [36], 2011, Sweden	Cohort study	[*n* = 374] IG = 184 CG = 170	Mendelson's maneuver Exercises and stretching of muscles of the head and neck	EORTC‐QLQ‐C30 EORTC‐H&N35	Global health status 56.6 (23.4) vs. 61.4 (23.0) (*p* = 0.29)	+++/Low
Van der Molen et al. [34], 2010, Netherlands	RCT	[*n* = 374] IG = 49 CG = 51	Range‐of‐motion exercises and three strengthening exercises (the effortful swallow, the Masako maneuver, and the super‐supraglottic swallow) Passive and slow opening of the mouth using specific device	QoL questionnaire (not validated)^a^	Patients perceived a significantly smaller mouth opening after treatment than before the onset of treatment (*p* = 0.032). No differences between the pretreatment and post‐treatment answers on the swallowing and information subscales were found	+++/Low

Abbreviations: CG, control group; CI, confidence interval; DAHANCA, Danish Head and Neck Cancer Group; DHI, Dysphagia Handicap Index; EORTC‐H&N35, EORTC quality of life Questionnaire Head and Neck (H&N)‐35 scores; EORTC QLQ‐C30, EORTC QLG Core Questionnaire; FOIS, Functional Oral Intake Scale; IG, intervention group; MDADI, The M.D. Anderson Dysphagia Inventory; *p*, *p* value; QoL, quality of life; RCT, randomized controlled trial; VAS, Visual Analog Scale; VHI, Voice Handicap Index; vs, versus.

^a^ Specific questionnaires about swallowing problems related to QoL; Quality/Bias according to JBI critical Appraisal Tools: High = +++; Medium = ++; Low = +.

### 3.2 Prehabilitation Interventions Identified

#### 3.2.1 Exercise and Stretching for Mobility and Trismus Prevention

A crucial step in patient preparation prior to radiotherapy was the consultation with a physiotherapist. The primary goal of this consultation was to preserve the mobility of areas targeted for radiation exposure, with a specific focus on the head and neck region. Through both oral and written communication, patients were provided with a regimen of exercises and muscle stretching, emphasizing active head movements such as rotations, flexions, and extensions, aimed at preventing neck stiffness [36]. A key aspect of this regimen was the inclusion of specific exercises to prevent the onset of trismus, a condition characterized by restricted jaw opening. To address this issue, the “Medic Jaw Trainer and Stretcher” (JTS) device was introduced, designed to maintain jaw mobility. Results showed that patients who adhered to this exercise and stretching program exhibited significant improvements in the mobility of areas exposed to radiotherapy. Additionally, there was a notable reduction in neck stiffness, and the prevention of trismus was effectively achieved, a common complication in patients undergoing radiation therapy. The use of the JTS device proved particularly beneficial in reducing the incidence of this condition.

#### 3.2.2 Home‐Based Training Programs With Periodic Oversight

Personal responsibility and adherence to a consistent home exercise routine played a pivotal role in patient recovery and overall well‐being. Approximately 2 weeks prior to the start of radiotherapy, participants were instructed in a series of exercises designed to be performed daily, with each session lasting approximately 10 min. Understanding the significant impact and necessity of these exercises was considered fundamental. To this end, patients were given thorough explanations to emphasize their importance. Additionally, exercise diaries were provided to serve both as a progress log and a self‐assessment tool [29]. Another aspect of the protocol highlighted the importance of periodic sessions supervised by a physiotherapist, focusing particularly on lymphedema self‐drainage techniques. Moreover, an additional intervention encouraged patients to chew sugar‐free gum as part of their daily regimen [33]. Patient adherence to the exercise regimen led to notable improvements in the ability to perform daily activities with reduced discomfort and pain. The exercise diaries also provided valuable insights into patient compliance with the program, which was generally high.

#### 3.2.3 Comprehensive Programs With Supervised Physiotherapy Sessions

The integration of structured physiotherapy sessions with routine patient care provided a comprehensive framework for enhancing recovery. Within the context of an RCT [33], patients were divided into two groups: one receiving specialized exercise‐based interventions and the other receiving standard care. Patients in the exercise group participated in tailored physiotherapy sessions, which included self‐administered lymph drainage techniques, customized exercises, and dietary aids such as sugar‐free gum. The underlying hypothesis was that combining physical exercise with nutritional guidance would significantly enhance postradiotherapy recovery. This holistic approach led to improved patient outcomes, particularly in reducing lymphedema. The synergy between physical exercise and dietary recommendations contributed to a notable improvement in the QoL for patients following radiation therapy.

#### 3.2.4 Jaw Mobility Programs

Jaw mobility and function were identified as critical areas of concern, addressed through a carefully structured 10‐week exercise program. This program emphasized not only repetitive exercises but also a progressive increase in intensity, ensuring both patient safety and comfort. Patients used specific jaw mobilization devices, engaging in a series of movements and stretches aimed at optimizing jaw mobility. A gradual increase in exercise intensity was a key aspect of the program, minimizing the risk of injury while ensuring effective results [34]. The adoption of this program led to significant improvements in jaw mobility and functionality, with participants experiencing reduced pain and a greater range of movement.

#### 3.2.5 Preventive Rehabilitation

The exploration of preventive measures was at the heart of a randomized rehabilitation trial. Two arms, termed standard and experimental, were investigated. While the standard rehabilitation program focused on a series of general exercises, the experimental one included stretch exercises utilizing specific devices. Participants were equipped with specialized tools and techniques to strengthen the muscles essential for swallowing, offering a dual approach to enhance mobility and ensure optimal swallowing function [34]. This trial highlighted that prehabilitation interventions can significantly impact the prevention of dysphagia and other side effects associated with radiation therapy. Participants in the experimental arm reported fewer swallowing issues and an improved QoL compared to the control group. The second part of the study [32] assessed the long‐term effects of two swallowing rehabilitation programs in 29 advanced head and neck cancer patients undergoing chemoradiotherapy (CRT). Patients were randomized into a standard group and an experimental group. Functional changes were evaluated at four time points using multidimensional measures. Many initial issues decreased significantly within the first year post‐treatment, except for xerostomia (59%). At 2 years, significant weight gain was observed (*p* = 0.000), mainly in the experimental group (*p* = 0.002) and in patients with disease below the hyoid bone. Both programs showed good compliance and similar outcomes, with slight advantages for the experimental group.

### 3.3 QoL

The studies analyzed assessed QoL using the EORTC‐QLQ‐C30, EORTC‐H&N35 [29, 30, 31, 33, 35, 36], or specific questionnaires related to swallowing problems and their impact on QoL [32, 34]. A retrospective study [29] of 60 advanced head and neck squamous cell carcinoma (HNSCC) patients treated with RT/CRT found that 71% reported significant swallowing dysfunction, impacting their QoL. Patients with hypopharyngeal and laryngeal cancers had better swallowing function and QoL compared to those with oral cavity or oropharyngeal tumors (*p* < 0.05), showing a strong correlation between dysphagia and voice problems (*p* < 0.05). Subsequently, in the second part of this study, prophylactic swallowing exercises led to significantly better post‐treatment MD Anderson Dysphagia Inventory (MDADI) (*p* = 0.006) and Dysphonia Handicap Index (DHI) scores (*p* = 0.002), improving QoL. A RCT [31] assessed the impact of prophylactic swallowing exercises on swallowing‐related outcomes in head and neck cancer patients undergoing curative RT. The study demonstrated a significant improvement in global health‐related QoL scores measured with the EORTC QLQ‐C30, with the intervention group showing slightly higher scores compared to the control group at 3 months (*p* = 0.03) and 5 months post‐treatment (*p* = 0.05). Similarly, another RCT [30] investigating the impact of preventive swallowing exercises during CRT measured QoL using the EORTC QLQ‐C30 and QLQ‐H&N35 scales. The results showed significant differences between the intervention and control groups for global health (score difference: 10.1, 95% CI, −0.1 to 20.3; *p* = 0.05) and social eating (score difference: −12.8, 95% CI, −25.8 to 0.2; *p* = 0.05) at 3 months post‐treatment. Another study [35] echoed these findings, showing no significant baseline differences in EORTC QLQ‐C30 scores. However, after 3 months, the intervention group demonstrated a significant improvement in global health (+12.2 vs. −0.8 in the control, *p* < 0.001), underscoring the potential impact of targeted therapeutic programs. In contrast to previous findings, a prospective longitudinal cohort study [36] evaluated the efficacy of various exercises, including Mendelson's maneuver and head and neck muscle stretching, in preventing reduced mobility and trismus. However, the study did not show significant improvements in QoL, as measured by the EORTC QLQ‐C30 global health status at 6 months (*p* = 0.29). Similarly, another study [33] involving 97 patients with head and neck cancer undergoing radiotherapy randomized participants into two groups: one group performed exercises supervised by a physiotherapist (*n* = 50) 5–6 times for 45 min during and after radiotherapy, in addition to standard care, while the other group received standard care alone. The exercise intervention did not result in any significant improvements in health‐related QoL (HRQoL) or in alleviating cancer‐related symptoms at 5 and 12 months of follow‐up.

Two additional studies also evaluated QoL using specific questionnaires focusing on swallowing function and mouth opening after CRT. One study [32] assessed the effect of preventive rehabilitation on these outcomes, showing improvements in QoL for swallowing (*p* = 0.08) and mouth opening (*p* = 0.03). Similarly, another prospective clinical trial [34] investigated the long‐term effects of two preventive swallowing rehabilitation programs in advanced head and neck cancer patients. The trial involved 29 patients randomized into two groups: a standard exercise group (S) and an experimental group (E) using the TheraBite Jaw Motion Rehabilitation System. At baseline, 36% of patients reported problems with swallowing liquids and 17% had xerostomia. Xerostomia increased significantly to 69% at 10 weeks (*p* = 0.001), remaining at 69% after 1 year (*p* = 0.000) and decreasing to 59% at 2 years (*p* = 0.004).

## 4 Discussion

Head and neck cancer is a complex medical condition that not only imposes significant physical challenges on patients but also profoundly impacts their QoL. This review aimed to evaluate the impact of physical prehabilitation interventions for patients undergoing radiotherapy or chemotherapy, focusing on improving patient outcomes and QoL during and after treatment.

### 4.1 Physical Prehabilitation Interventions

The findings regarding physical prehabilitation interventions indicate that patients who participated in supervised exercise programs experienced several clinical benefits, including reduced fatigue and improved tolerance to oncological treatments [29, 33, 34, 36]. These results are consistent with several international studies investigating the efficacy of prehabilitation in various oncological contexts. For instance, a study on lung cancer patients reported that prehabilitation exercises significantly reduced postoperative recovery times and improved functional capacity [37, 38]. Similarly, research involving patients with esophageal and gastric cancers [39] demonstrated that integrating physical prehabilitation improved functional capacity both before and after surgery. Additionally, an analysis conducted on patients with bladder cancer supported the effectiveness of prehabilitation in improving various physical and nutritional outcomes postsurgery, particularly by reducing muscle mass loss [40].

### 4.2 Impact on QoL

A critical aspect examined in this review is the impact of prehabilitation on QoL. The results showed a significant improvement in QoL, as measured by the EORTC QLQ‐C30, EORTC QLQ‐H&N35 questionnaires [29, 30, 31, 33, 35, 36], and specific questionnaires assessing swallowing problems related to QoL [32, 34]. Patients reported improvements in physical function, psychological well‐being, and their ability to perform daily activities. These findings are supported by international studies that demonstrate the positive impact of prehabilitation on patients with both oncological and nononcological conditions. For instance, a meta‐analysis [41] revealed that prehabilitation programs in cancer patients led to significant improvements in postoperative QoL and functional capacity. Similarly, a recent Cochrane review [42] involving colon cancer patients confirmed that prehabilitation enhances functional capacity, further underscoring its efficacy.

When considering the effectiveness of prehabilitation in nononcological diseases and fragile patient populations, similar improvements in functional well‐being and perceived QoL have been observed. Specifically, studies on patients with cardiovascular diseases [43] reported that prehabilitation improved perioperative heart rate and preoperative QoL in patients awaiting coronary artery bypass graft surgery. Another review [44] demonstrated that prehabilitation positively affected postoperative complications and functional recovery in frail older patients, showing a significant reduction in total complications (*p* = 0.021). However, no significant differences were found in mortality (*p* = 0.176), readmission rates (*p* = 0.906), or length of stay (*p* = 0.540).

These findings highlight the importance of physical prehabilitation, which is already a core component of Enhanced Recovery After Surgery (ERAS) protocols. Our results, particularly in the context of head and neck cancer patients undergoing chemotherapy and radiotherapy, reinforce the growing significance of prehabilitation in improving clinical outcomes [45]. This underscores its increasing relevance in oncological settings, where integrating prehabilitation before treatment shows promising potential to enhance both preoperative and postoperative recovery, ultimately contributing to a better QoL for patients. Our findings, consistent with international literature on head and neck cancer patients [17, 46], suggest that prehabilitation is an effective intervention for improving the QoL of cancer patients, offering benefits that extend both physically and psychologically.

However, despite these positive outcomes, it is essential to consider the variability in the effectiveness of prehabilitation across studies. Not all research has demonstrated significant improvements in QoL or functional outcomes. For example, a study by Høgdal et al. [33] showed that supervised physiotherapy exercises did not lead to significant improvements in health‐related QoL or alleviation of cancer‐related symptoms during follow‐up. Similarly, Ahlberg et al. [36] found no significant improvement in global health status as measured by the EORTC QLQ‐C30 at 6 months' post‐treatment (*p* = 0.29). These inconsistencies suggest that while prehabilitation interventions may benefit certain aspects of recovery, such as preventing trismus or improving swallowing function, the overall impact can be influenced by factors like the type of intervention, patient characteristics, and the specific outcomes measured. Therefore, while the growing body of evidence supports the potential benefits of prehabilitation, it is crucial to recognize that not all interventions yield uniformly positive results. This variability highlights the need for further research to identify the most effective conditions and intervention strategies, ensuring that prehabilitation is tailored to meet the specific needs of individual patients. In particular, future studies should focus on determining which patient parameters, such as age, cancer stage, comorbidities, and baseline physical function, are most predictive of success with prehabilitation. Additionally, it is essential to explore the barriers and facilitators to implementing prehabilitation on a wider scale. Key barriers may include a lack of specialized expertise, inadequate resources for delivering interventions, and logistical challenges related to the centralization of care. On the other hand, facilitators such as multidisciplinary team collaboration, the integration of prehabilitation into existing clinical pathways, and the availability of technology to support remote or home‐based training could help overcome these challenges [47, 48]. To implement prehabilitation widely in clinical practice, it will be necessary to develop clear guidelines and frameworks that address these issues and ensure that interventions are accessible, effective, and sustainable for all patients, regardless of their specific circumstances.

### 4.3 Limitations

The present study is subject to certain limitations. The primary limitation of this scoping review is the heterogeneity among the included studies, study participants, outcome measures, and follow‐up duration. Additional limitations include the variation and variability in oncological treatments. Furthermore, this review did not specifically examine any particular cancer site or severity to determine whether these factors influenced the efficacy of prehabilitation. It was not possible to aggregate the data for a pooled analysis. Lastly, it is possible that the rigorous search protocol and imposed limitations may have excluded other relevant studies on this topic, including those published in languages other than English.

## 5 Conclusions

Prehabilitation interventions represent a comprehensive approach to enhancing patient outcomes and QoL. Evaluating QoL in individuals receiving treatment for head and neck cancer is an essential component of their healthcare, as it enables providers to understand the effects of treatment on various aspects of overall well‐being. Physical prehabilitation and psychological support play crucial roles in determining patients' well‐being. While some interventions demonstrate clear benefits, the impact on overall QoL varies across studies. Tailoring interventions based on patient characteristics and treatment modalities may lead to more effective improvements in QoL. Further research is needed to refine these approaches and enhance the overall QoL of head and neck cancer survivors.

## Author Contributions

A.M.S.: conceptualization, methodology, writing original draft, review and editing, investigation, visualization; M.P.: methodology, review and editing, investigation, visualization; G.C.: writing original draft, review and editing, investigation; S.M.P.: writing original draft, review and editing, investigation; S.M.: conceptualization, methodology, writing original draft, review and editing, investigation, visualization; coordinator; G.D.P.: methodology, review and editing, visualization, coordinator; F.P.: review and editing, visualization; M.S.: review and editing, investigation, visualization, coordinator; B.M.: review and editing, investigation, visualization, coordinator. A.M.S. and M.P. provided an equal contribution as first author in drafting the manuscript; M.S. and B.M. provided an equal contribution as last author to the coordination of the research group. All authors read and approved the final manuscript.

## Funding

This work was supported by BIBLIOSAN.

## Conflicts of Interest

The authors declare no conflicts of interest.

## Supporting information


**Table S1:** Search Strategy.
**Table S2:** JBI Critical appraisal tool for randomized controlled trials.
**Table S3:** JBI Critical appraisal tool for case control studies.
**Table S4:** Critical appraisal of cohort studies included.
**Table S5:** JBI Critical appraisal tool of analytical cross‐sectional studies.

## Data Availability

All data are provided within the manuscript and in the Supporting Information.

## References

[1] E. E. W. Cohen , S. J. LaMonte , N. L. Erb , et al., “American Cancer Society Head and Neck Cancer Survivorship Care Guideline: ACS HNC Survivorship Care Guideline,” CA: A Cancer Journal for Clinicians 66, no. 3 (2016): 203–239, 10.3322/caac.21343. 27002678

[2] N. Zahid , R. S. Martins , Z. S. Dawood , et al., “Clinical and Psychosocial Factors Associated With Quality of Life in Patients With Head and Neck Cancer: An Analytical Cross‐Sectional Study From a Lower‐Middle‐Income Country,” BMC Psychol 11, no. 1 (2023): 265, 10.1186/s40359-023-01264-6. 37670380 PMC10478451

[3] A. M. Santagostino , D. Cannizzaro , F. Soekeland , S. Mancin , and B. Mazzoleni , “Pain and Quality of Life in Patients Undergoing Lumbar Arthrodesis for Degenerative Spondylolisthesis: A Systematic Review,” World Neurosurgery 177 (2023): 172–183.e12, 10.1016/j.wneu.2023.06.047. 37348603

[4] Y. Sharma , G. Mishra , and V. Parikh , “Quality of Life in Head and Neck Cancer Patients,” Indian Journal of Otolaryngology and Head & Neck Surgery 71, no. S1 (2019): 927–932, 10.1007/s12070-019-01620-2. 31742096 PMC6848337

[5] Y. S. Wu , P. Y. Lin , C. Y. Chien , et al., “Anxiety and Depression in Patients With Head and Neck Cancer: 6‐Month Follow‐Up Study,” Neuropsychiatric Disease and Treatment 12 (2016): 1029–1036, 10.2147/NDT.S103203. 27175080 PMC4854266

[6] A. Lewandowska , G. Rudzki , T. Lewandowski , et al., “Quality of Life of Cancer Patients Treated With Chemotherapy,” International Journal of Environmental Research and Public Health 17, no. 19 (2020): 6938, 10.3390/ijerph17196938. 32977386 PMC7579212

[7] İ. Altun and A. Sonkaya , “The Most Common Side Effects Experienced by Patients Were Receiving First Cycle of Chemotherapy,” Iran J Public Health 47, no. 8 (2018): 1218–1219, http://ijph.tums.ac.ir. 30186799 PMC6123577

[8] S. Hitomi , I. Ujihara , M. Sago‐Ito , et al., “Hyposalivation due to Chemotherapy Exacerbates Oral Ulcerative Mucositis and Delays Its Healing,” Archives of Oral Biology 105 (2019): 20–26, 10.1016/j.archoralbio.2019.06.003. 31238198

[9] Y. Iijima , M. Yamada , M. Endo , et al., “Dysgeusia in Patients With Cancer Undergoing Chemotherapy,” Journal of Oral and Maxillofacial Surgery, Medicine, and Pathology 31, no. 3 (2019): 214–217, 10.1016/j.ajoms.2019.01.006.

[10] B. Mazzoleni , G. Ferrari , F. Savioni , et al., “Non‐Pharmacological Strategies to Alleviate Dysgeusia in Patients Undergoing Chemotherapy: A Systematic Review,” European Journal of Oncology Nursing 70 (2024): 102569, 10.1016/j.ejon.2024.102569. 38593535

[11] G. De Pasquale , S. Mancin , S. Matteucci , et al., “Nutritional Prehabilitation in Head and Neck Cancer: A Systematic Review of Literature,” Clinical Nutrition ESPEN 58 (2023): 326–334, 10.1016/j.clnesp.2023.10.033. 38057023

[12] F. Bozzetti and P. Cotogni , “Nutritional Issues in Head and Neck Cancer Patients,” Healthcare (Basel) 8, no. 2 (2020): 102, 10.3390/healthcare8020102. 32316416 PMC7348698

[13] T. Powrózek , J. Dziwota , and T. Małecka‐Massalska , “Nutritional Deficiencies in Radiotherapy‐Treated Head and Neck Cancer Patients,” Journal of Clinical Medicine 10, no. 4 (2021): 574, 10.3390/jcm10040574. 33546506 PMC7913750

[14] M. Rucińska and K. Osowiecka , “Prehabilitation as an Extra Approach to Usual Care for Cancer Patients,” Nowotwory. Journal of Oncology 72, no. 5 (2022): 294–302, 10.5603/NJO.a2022.0036.

[15] R. Crevenna , S. Palma , and T. Licht , “Cancer Prehabilitation—A Short Review,” Memo – Magazine of European Medical Oncology 14, no. 1 (2021): 39–43, 10.1007/s12254-021-00686-5.

[16] D. Santa Mina , S. J. Van Rooijen , E. M. Minnella , et al., “Multiphasic Prehabilitation Across the Cancer Continuum: A Narrative Review and Conceptual Framework,” Frontiers in Oncology 10 (2021): 598425, 10.3389/fonc.2020.598425. 33505914 PMC7831271

[17] I. Loewen , C. C. Jeffery , J. Rieger , and G. Constantinescu , “Prehabilitation in Head and Neck Cancer Patients: A Literature Review,” Journal of Otolaryngology – Head & Neck Surgery 50, no. 1 (2021): 2, 10.1186/s40463-020-00486-7. 33407922 PMC7789666

[18] V. Srinivas , U. Choubey , J. Motwani , et al., “Synergistic Strategies: Optimizing Outcomes Through a Multidisciplinary Approach to Clinical Rounds,” Proceedings (Baylor University Medical Center) 37, no. 1 (2023): 144–150, 10.1080/08998280.2023.2274230. 38174031 PMC10761132

[19] C. Dolislager , K. Polo , and T. Wickboldt , “A Comprehensive Prehabilitative Model of Care for Head and Neck Cancer Survivors: A Scoping Review,” Archives of Physical Medicine and Rehabilitation 103, no. 12 (2022): e201, 10.1016/j.apmr.2022.08.031.

[20] R. Brady , L. McSharry , S. Lawson , and J. Regan , “The Impact of Dysphagia Prehabilitation on Swallowing Outcomes Post‐Chemoradiation Therapy in Head and Neck Cancer: A Systematic Review,” European Journal of Cancer Care (Engl) [Internet] 31, no. 3 (2022): e13549, 10.1111/ecc.13549. 34964185

[21] University of Calgary , “Multiphasic Prehabilitation in Patients Undergoing Surgery for Head and Neck Cancer [Internet],” clinicaltrials.gov; 2022 Feb [cited 2023 Jan 1]. Report No.: NCT04598087, https://clinicaltrials.gov/study/NCT04598087.

[22] Associacao de Investigacao de Cuidados de Suporte em Oncologia , Exercise Prehabilitation in Patients With Head and Neck Squamous‐Cell Carcinoma: The FIT4TREAT Trial [Internet] clinicaltrials.gov; 2023 Jul [cited 2023 Jan 1]. Report No.: NCT05418842, https://clinicaltrials.gov/study/NCT05418842.

[23] M. D. J. Peters , C. Marnie , A. C. Tricco , et al., “Updated Methodological Guidance for the Conduct of Scoping Reviews,” JBI Evidence Synthesis 18 (2020): 2119–2126, 10.11124/JBIES-20-00167. 33038124

[24] Z. Munn , D. Pollock , H. Khalil , et al., “What Are Scoping Reviews? Providing a Formal Definition of Scoping Reviews as a Type of Evidence Synthesis,” JBI Evidence Synthesis 20, no. 4 (2022): 950–952, 10.11124/JBIES-21-00483. 35249995

[25] A. C. Tricco , E. Lillie , W. Zarin , et al., “PRISMA Extension for Scoping Reviews (PRISMA‐ScR): Checklist and Explanation,” Annals of Internal Medicine 169 (2018): 467–473, 10.7326/M18-0850. 30178033

[26] D. Levac , H. Colquhoun , and K. K. O'Brien , “Scoping Studies: Advancing the Methodology,” Implementation Science 5, no. 1 (2010): 69, 10.1186/1748-5908-5-69. 20854677 PMC2954944

[27] M. D. Peters , C. M. Godfrey , H. Khalil , P. McInerney , D. Parker , and C. B. Soares , “Guidance for Conducting Systematic Scoping Reviews,” International Journal of Evidence‐Based Healthcare 13, no. 3 (2015): 141–146, 10.1097/XEB.0000000000000050. 26134548

[28] M. Sguanci , G. Ferrara , S. M. Palomares , et al., “Dysgeusia and Chronic Kidney Disease: A Scoping Review,” Journal of Renal Nutrition 34, no. 5 (2024): 374–390, 10.1053/j.jrn.2024.04.005. 38729584

[29] I. Carmignani , L. G. Locatello , I. Desideri , et al., “Analysis of Dysphagia in Advanced‐Stage Head‐And‐Neck Cancer Patients: Impact on Quality of Life and Development of a Preventive Swallowing Treatment,” European Archives of Oto‐Rhino‐Laryngology 275, no. 8 (2018): 2159–2167, 10.1007/s00405-018-5054-9. 29978259

[30] B. P. Pisano Messing , E. C. Ward , C. L. Lazarus , et al., “Prophylactic Swallow Therapy for Patients With Head and Neck Cancer Undergoing Chemoradiotherapy: A Randomized Trial,” Dysphagia 32, no. 4 (2017): 487–500, 10.1007/s00455-017-9790-6. 28444488 PMC5515964

[31] H. R. Mortensen , K. Jensen , K. Aksglæde , K. Lambertsen , E. Eriksen , and C. Grau , “Prophylactic Swallowing Exercises in Head and Neck Cancer Radiotherapy,” Dysphagia 30, no. 3 (2015): 304–314, 10.1007/s00455-015-9600-y. 25690840

[32] L. van der Molen , M. A. van Rossum , C. R. Rasch , L. E. Smeele , and F. J. Hilgers , “Two‐Year Results of a Prospective Preventive Swallowing Rehabilitation Trial in Patients Treated With Chemoradiation for Advanced Head and Neck Cancer,” European Archives of Oto‐Rhino‐Laryngology 271, no. 5 (2014): 1257–1270, 10.1007/s00405-013-2640-8. 23892729

[33] N. Høgdal , C. Juhl , M. Aadahl , and C. Gluud , “Early Preventive Exercises Versus Usual Care Does Not Seem to Reduce Trismus in Patients Treated With Radiotherapy for Cancer in the Oral Cavity or Oropharynx: A Randomised Clinical Trial,” Acta Oncologica 54, no. 1 (2015): 80–87, 10.3109/0284186X.2014.954677. 25229260

[34] L. Van Der Molen , M. A. Van Rossum , L. M. Burkhead , L. E. Smeele , C. R. N. Rasch , and F. J. M. Hilgers , “A Randomized Preventive Rehabilitation Trial in Advanced Head and Neck Cancer Patients Treated With Chemoradiotherapy: Feasibility, Compliance, and Short‐Term Effects,” Dysphagia 26, no. 2 (2011): 155–170, 10.1007/s00455-010-9288-y. 20623305 PMC3098976

[35] N. Pauli , B. Fagerberg‐Mohlin , P. Andréll , and C. Finizia , “Exercise Intervention for the Treatment of Trismus in Head and Neck Cancer,” Acta Oncologica 53, no. 4 (2014): 502–509, 10.3109/0284186X.2013.837583. 24175896

[36] A. Ahlberg , T. Engström , P. Nikolaidis , et al., “Early Self‐Care Rehabilitation of Head and Neck Cancer Patients,” Acta Oto‐Laryngologica 131, no. 5 (2011): 552–561, 10.3109/00016489.2010.532157. 21492066 PMC3082166

[37] K. Wade‐Mcbane , A. King , C. Urch , J. Jeyasingh‐Jacob , A. Milne , and C. L. Boutillier , “Prehabilitation in the Lung Cancer Pathway: A Scoping Review,” BMC Cancer 23, no. 1 (2023): 747, 10.1186/s12885-023-11254-x. 37568130 PMC10416419

[38] C. M. Michael , E. J. Lehrer , K. H. Schmitz , and N. G. Zaorsky , “Prehabilitation Exercise Therapy for Cancer: A Systematic Review and Meta‐Analysis,” Cancer Medicine 10, no. 13 (2021): 4195–4205, 10.1002/cam4.4021. 34110101 PMC8267161

[39] E. M. Minnella , R. Awasthi , S. E. Loiselle , R. V. Agnihotram , L. E. Ferri , and F. Carli , “Effect of Exercise and Nutrition Prehabilitation on Functional Capacity in Esophagogastric Cancer Surgery: A Randomized Clinical Trial,” JAMA Surgery 153, no. 12 (2018): 1081–1089, 10.1001/jamasurg.2018.1645. 30193337 PMC6583009

[40] S. Dal Bello , S. Mancin , S. Morales Palomares , et al., “Nutritional Prehabilitation in Patients Undergoing Cystectomy: A Systematic Review,” Nutrients 16, no. 11 (2024): 1682, 10.3390/nu16111682. 38892615 PMC11174884

[41] Y. J. Chou , H. J. Kuo , and S. C. Shun , “Cancer Prehabilitation Programs and Their Effects on Quality of Life,” Oncology Nursing Forum 45, no. 6 (2018): 726–736, 10.1188/18.ONF.726-736. 30339146

[42] C. J. Molenaar , S. J. van Rooijen , H. J. Fokkenrood , R. M. Roumen , L. Janssen , and G. D. Slooter , “Prehabilitation Versus no Prehabilitation to Improve Functional Capacity, Reduce Postoperative Complications and Improve Quality of Life in Colorectal Cancer Surgery,” Cochrane Database of Systematic Reviews 5, no. 5 (2022): CD013259, 10.1002/14651858.CD013259.pub2. 35588252 PMC9118366

[43] C. Steinmetz , B. Bjarnason‐Wehrens , H. Baumgarten , T. Walther , T. Mengden , and C. Walther , “Prehabilitation in Patients Awaiting Elective Coronary Artery Bypass Graft Surgery – Effects on Functional Capacity and Quality of Life: A Randomized Controlled Trial,” Clinical Rehabilitation 34, no. 10 (2020): 1256–1267, 10.1177/0269215520933950. 32546065 PMC7477776

[44] K. Y. She , L. Huang , H. T. Zhang , et al., “Effect of Prehabilitation on Postoperative Outcomes in the Frail Older People: A Systematic Review and Meta‐Analysis,” Geriatric Nursing 55 (2024): 79–88, 10.1016/j.gerinurse2023.10.027. 37976559

[45] M. L. Rasmussen , S. G. Leeds , E. P. Whitfield , B. Aladegbami , G. O. Ogola , and M. A. Ward , “Enhanced Recovery After Surgery (ERAS) Decreases Complications and Reduces Length of Stay in Foregut Surgery Patients,” Surgical Endoscopy 37, no. 4 (2023): 2842–2850, 10.1007/s00464-022-09806-6. 36481822

[46] I. Seth , G. Bulloch , K. R. Qin , et al., “Pre‐Rehabilitation Interventions for Patients With Head and Neck Cancers: A Systematic Review and Meta‐Analysis,” Head & Neck 46, no. 1 (2024): 86–117, 10.1002/hed.27561. 37897197

[47] I. Phillips , C. McDougall , A. Walton , et al., “Multidisciplinary Prehabilitation Reduces Hospitalization Time and Suggests Improved Survival in Patients with Radiologically Diagnosed Lung Cancer,” Cancers 17, no. 20 (2025): 3329, 10.3390/cancers17203329. 41154388 PMC12562606

[48] M. Sguanci , S. Mancin , M. Piredda , et al., “Nursing‐Engineering Interdisciplinary Research: A Synthesis of Methodological Approach to Perform Healthcare‐Technology Integrated Projects,” MethodsX 12 (2023): 102525, 10.1016/j.mex.2023.102525. 38204982 PMC10776977

