# Supplementary material for: Enhancing Quality of Life in Head and Neck Cancer: A Scoping Review on the Role of Physical Prehabilitation
Source: Cancer Med. 2026 Mar 27;15(4):e71743. doi: 10.1002/cam4.71743 (PMC13140588; doi:10.1002/cam4.71743)
Supplement: Supplementary file 1 — Table S1: Search Strategy. Table S2: JBI Critical appraisal tool for randomized controlled trials. Table S3: JBI Critical appraisal tool for case control studies. Table S4: Critical appraisal of cohort studies included. Table S5: JBI Critical appraisal tool of analytical cross‐sectional studies. [file CAM4-15-e71743-s001.doc]

**Table S1.** Search Strategy

| **ID** | **Cochrane Library** | **Results** |
| --- | --- | --- |
| #1 | radiotherapy OR radiotherapies OR "radiation therapy" OR "radiation therapies" OR "radiation treatment" "radiation treatments" OR "radiation Therapies" OR "radiation Therapy" OR chemotherapies OR chemotherapy |
| #2 | "Neoplasms Head and Neck" OR "Head and Neck Neoplasm" OR "Head and Neck Cancer" OR "Head and Neck Cancers" OR "Neck Upper Neoplasm*" OR "Neck Upper Cancer*" OR "UADT Neoplasm*" OR "Head Neoplasm*" OR "Head cancer*" OR "Neck Neoplasm*" OR "Neck cancer*" |
| #3 | Deglutition OR Deglutitions OR Swallowing OR Swallowings OR "Deglutition disorder*" OR "Deglutitions disorder*" OR "Swallowing disorder*" OR "Swallowings disorder*" OR nutrition OR nutritional OR dysphagia |
| #4 | Prehabilitation OR Prehabilitations OR Pre-habilitation OR Pre-habilitations OR "exercise prehabilitation*" OR "exercise pre-habilitation*" OR "exercises prehabilitation*" OR "exercises pre-habilitation*" OR "prehabilitation Conditioning" OR "prehabilitation Conditioning" |
| **#5** | **#1 AND #2 AND #3 AND #4** | **5** |
| #6 | MeSH descriptor: [Radiotherapy] explode all trees |  |
| #7 | MeSH descriptor: [Drug Therapy] explode all trees |  |
| #8 | MeSH descriptor: [Antineoplastic Agents] explode all trees |  |
| #9 | MeSH descriptor: [Head and Neck Neoplasms] explode all trees |  |
| #10 | MeSH descriptor: [Malnutrition] explode all trees and with qualifier(s): [Prevention and control – PC] |  |
| **#11** | **#6 AND #7 AND #8 AND #9 AND #10**  **#1 AND #2 AND #10** | **0**  **6** |
| **#12** | **#9 AND #10** | **19** |
| #13 | “Nutritional prehabilitation“ |  |
| #14 | Head and neck cancer |  |
| **#15** | **#13 AND #14**  **#13 AND #9**  **#4 AND #2** | **0**  **1**  **12** |
| #16 | Early nutritional support |  |
| #17 | MeSH descriptor: [Head and Neck Neoplasms] explode all trees |  |
| **#18** | **#16 AND #17** | **28** |
| **#19** | **#16 AND #2** | **35** |
| **#20** | **#3 AND #2 AND #4** | **6** |
| #21 | MeSH descriptor: [Deglutition Disorders] explode all trees and with qualifier(s): [Prevention and control – PC] |  |
| #22 | "Neoplasms Head and Neck" OR "Head and Neck Neoplasm" OR "Head and Neck Cancer" OR "Head and Neck Cancers" OR "Neck Upper Neoplasm*" OR "Neck Upper Cancer*" OR "UADT Neoplasm*" OR "Head Neoplasm*" OR "Head cancer*" OR "Neck Neoplasm*" OR "Neck cancer*" |  |
| **#23** | **#21 AND #22** | **26** |
| #24 | "Quality of life" OR "Life Quality" OR "Health-Related Quality Of Life" OR "Health Related Quality Of Life" OR "HRQOL" OR "EORTC QLQ" |  |
| **#25** | **#1 AND #2 AND #4 AND #24** | **5** |
| **#26** | **#1 OR #2 AND #4 AND #24** | **658** |
| **ID** | **Pubmed** | **Results** |
| #1 | radiotherapy OR radiotherapies OR "radiation therapy" OR "radiation therapies" OR "radiation treatment" "radiation treatments" OR "radiation Therapies" OR "radiation Therapy" OR chemotherapies OR chemotherapy |
| #2 | "Neoplasms Head and Neck" OR "Head and Neck Neoplasm" OR "Head and Neck Cancer" OR "Head and Neck Cancers" OR "Neck Upper Neoplasm*" OR "Neck Upper Cancer*" OR "UADT Neoplasm*" OR "Head Neoplasm*" OR "Head cancer*" OR "Neck Neoplasm*" OR "Neck cancer*" |
| #3 | Deglutition OR Deglutitions OR Swallowing OR Swallowings OR "Deglutition disorder*" OR "Deglutitions disorder*" OR "Swallowing disorder*" OR "Swallowings disorder*" OR nutrition OR nutritional OR dysphagia |
| #4 | Prehabilitation OR Prehabilitations OR Pre-habilitation OR Pre-habilitations OR "exercise prehabilitation*" OR "exercise pre-habilitation*" OR "exercises prehabilitation*" OR "exercises pre-habilitation*" OR "prehabilitation Conditioning" OR "prehabilitation Conditioning" |
| **#5** | **#1 AND #2 AND #3 AND #4** | **8** |
| #6 | (("Radiotherapy"[Mesh]) OR ("Induction Chemotherapy"[Mesh])) |  |
| #7 | ("Malnutrition/prevention and control"[Mesh]) |  |
| #8 | "Head and Neck Neoplasms"[Mesh] |  |
| **#9** | **#6 AND #7 AND #8**  **#1 AND #7 AND #2** | **23**  **28** |
| **#10** | **#7 AND #8** | **113** |
| #11 | “Nutritional prehabilitation” |  |
| #12 | head and neck cancer |  |
| **#13** | **#11 AND #12**  **#11 AND #8**  **#4 AND #2** | **4**  **4**  **47** |
| #14 | Early nutritional support |  |
| #15 | "Head and Neck Neoplasms"[Mesh] |  |
| **#16** | **#14 AND #15** | **334** |
| **#17** | **#14 AND #2** | **165** |
| **#18** | **#3 AND #2 AND #4** | **24** |
| #19 | ("Deglutition Disorders/prevention and control"[Mesh]) |  |
| #20 | "Neoplasms Head and Neck" OR "Head and Neck Neoplasm" OR "Head and Neck Cancer" OR "Head and Neck Cancers" OR "Neck Upper Neoplasm*" OR "Neck Upper Cancer*" OR "UADT Neoplasm*" OR "Head Neoplasm*" OR "Head cancer*" OR "Neck Neoplasm*" OR "Neck cancer*" |  |
| #21 | **#19 AND #20** | **118** |
| #22 | "Quality of life" OR "Life Quality" OR "Health-Related Quality Of Life" OR "Health Related Quality Of Life" OR "HRQOL" OR "EORTC QLQ" |  |
| **#23** | **#1 AND #2 AND #4 AND #22** | **9** |
| **#24** | **#1 OR #2 AND #4 AND #22** | **116** |
| **ID** | **Cumulative Index Of Nursing And Allied Health Literature (CINAHL)** | **Results** |
| #1 | radiotherapy OR radiotherapies OR "radiation therapy" OR "radiation therapies" OR "radiation treatment" "radiation treatments" OR "radiation Therapies" OR "radiation Therapy" OR chemotherapies OR chemotherapy |
| #2 | "Neoplasms Head and Neck" OR "Head and Neck Neoplasm" OR "Head and Neck Cancer" OR "Head and Neck Cancers" OR "Neck Upper Neoplasm*" OR "Neck Upper Cancer*" OR "UADT Neoplasm*" OR "Head Neoplasm*" OR "Head cancer*" OR "Neck Neoplasm*" OR "Neck cancer*" |
| #3 | Deglutition OR Deglutitions OR Swallowing OR Swallowings OR "Deglutition disorder*" OR "Deglutitions disorder*" OR "Swallowing disorder*" OR "Swallowings disorder*" OR nutrition OR nutritional OR dysphagia |
| #4 | Prehabilitation OR Prehabilitations OR Pre-habilitation OR Pre-habilitations OR "exercise prehabilitation*" OR "exercise pre-habilitation*" OR "exercises prehabilitation*" OR "exercises pre-habilitation*" OR "prehabilitation Conditioning" OR "prehabilitation Conditioning" |
| **#5** | **#1 AND #2 AND #3 AND #4** | **4** |
| #6 | (MH "Radiotherapy+") |  |
| #7 | (MH "Chemotherapy, Cancer+") |  |
| #8 | (MH "Malnutrition+/PC") |  |
| #9 | (MH "Head and Neck Neoplasms+") |  |
| **#10** | **#6 AND #7 AND #8 AND #9**  **#1 AND #2 AND #8** | **5**  **22** |
| **#11** | **#8 AND #9** | **54** |
| #12 | Nutritional prehabilitation |  |
| #13 | Head and neck cancer |  |
| **#14** | **#12 AND #13**  **#12 AND #9**  **#2 AND #4** | **1**  **0**  **24** |
| #15 | Early nutritional support |  |
| #16 | (MH "Head and Neck Neoplasms+") |  |
| **#17** | **#15 AND #16** | **2** |
| **#18** | **#15 AND #2** | **1** |
| **#19** | **#2 AND #3 AND #4** | **12** |
| #20 | (MH "Deglutition Disorders/PC") |  |
| #21 | "Neoplasms Head and Neck" OR "Head and Neck Neoplasm" OR "Head and Neck Cancer" OR "Head and Neck Cancers" OR "Neck Upper Neoplasm*" OR "Neck Upper Cancer*" OR "UADT Neoplasm*" OR "Head Neoplasm*" OR "Head cancer*" OR "Neck Neoplasm*" OR "Neck cancer*" |  |
| **#22** | **#20 AND #21** | **57** |
| #23 | "Quality of life" OR "Life Quality" OR "Health-Related Quality Of Life" OR "Health Related Quality Of Life" OR "HRQOL" OR "EORTC QLQ" |  |
| **#24** | **#1 AND #2 AND #4 AND #23** | **3** |
| **#25** | **#1 OR #2 AND #4 AND #23** | **5** |
| **#26** | **#2 AND #4** | **24** |
| **ID** | **EMBASE** |  |
| #1 | radiotherapy OR radiotherapies OR "radiation therapy" OR "radiation therapies" OR "radiation treatment" "radiation treatments" OR "radiation Therapies" OR "radiation Therapy" OR chemotherapies OR chemotherapy |
| #2 | "Neoplasms Head and Neck" OR "Head and Neck Neoplasm" OR "Head and Neck Cancer" OR "Head and Neck Cancers" OR "Neck Upper Neoplasm*" OR "Neck Upper Cancer*" OR "UADT Neoplasm*" OR "Head Neoplasm*" OR "Head cancer*" OR "Neck Neoplasm*" OR "Neck cancer*" |
| #3 | Deglutition OR Deglutitions OR Swallowing OR Swallowings OR "Deglutition disorder*" OR "Deglutitions disorder*" OR "Swallowing disorder*" OR "Swallowings disorder*" OR nutrition OR nutritional OR dysphagia |
| #4 | Prehabilitation OR Prehabilitations OR Pre-habilitation OR Pre-habilitations OR "exercise prehabilitation*" OR "exercise pre-habilitation*" OR "exercises prehabilitation*" OR "exercises pre-habilitation*" OR "prehabilitation Conditioning" OR "prehabilitation Conditioning" |
| **#5** | **#1 AND #2 AND #3 AND #4** | **19** |
| **#6** | **#2 AND #4** | **67** |
| #7 | 'nutritional prehabilitation' OR (nutritional AND ('prehabilitation'/exp OR prehabilitation)) |  |
| #8 | ('head'/exp OR head) AND ('neck cancer'/exp OR 'neck cancer' OR (('neck'/exp OR neck) AND ('cancer'/exp OR cancer))) |  |
| **#9** | **#7 AND #8** | **11** |
| #10 | 'dysphagia'/exp OR dysphagia |  |
| **#11** | **#10 AND #4 AND #2** | **17** |
| **#13** | **#1 OR #2 AND #3 AND #4** | **202** |

**Table S2.** JBI Critical appraisal tool for randomized controlled trials

| **STUDY** | **ITEM 1** | **ITEM 2** | **ITEM 3** | **ITEM 4** | **ITEM 5** | **ITEM 6** | **ITEM 7** | **ITEM 8** | **ITEM 9** | **ITEM 10** | **ITEM 11** | **ITEM 12** | **ITEM 13** | **INCLUDE** | **EXCLUDE** | **SEEK FURTHER INFO** | **SCORE (mean)** |
| --- | --- | --- | --- | --- | --- | --- | --- | --- | --- | --- | --- | --- | --- | --- | --- | --- | --- |
| Pisano Messing et al.,  2016 | Y | Y | Y | Y | Y | Y | Y | Y | Y | Y | Y | Y | Y | Y |  |  | 100% |
| Mortesen et al.2015 | Y | Y | Y | Y | Y | Y | Y | Y | Y | Y | Y | Y | Y | Y |  |  | 100% |
| Høgdal et al.,  2014 | Y | Y | Y | Y | Y | Y | Y | N | Y | Y | Y | Y | Y | Y |  |  | 92% |
| Van der Molen et al., 2013 | y | y | y | y | y | y | y | y | y | y | y | y | y | y |  |  | 100% |
| Van der Molen et al.,  2010 | Y | Y | Y | Y | Y | Y | Y | Y | Y | Y | Y | Y | Y | Y |  |  | 100% |

*Legend:* Y=Yes; N=No; U=Unclear; NA=Not Applicable / Items from Critical appraisal *tool for randomized controlled trials: 1= Was true randomization used for assignment of participants to treatment groups?; 2=Was allocation to treatment groups concealed?; 3=Were treatment groups similar at the baseline?; 4=Were participants blind to treatment assignment?; 5=Were those delivering treatment blind to treatment assignment? 6=Were outcomes assessors blind to treatment assignment?; 7=Were treatment groups treated identically other than the intervention of interest?; 8=Was follow up complete and if not, were differences between groups in terms of their follow up adequately described and analyzed?; 9=Were participants analyzed in the groups to which they were randomized?; 10=Were outcomes measured in the same way for treatment groups?*; *11=Were outcomes measured a reliable way?; 12=Was appropriate statistical analysis used?; 13=Was the trial design appropriate, and any deviations from the standard RCT design (individual randomization, parallel groups) accounted for in the conduct and analysis of the trial?*

**Table S3.** JBI Critical appraisal tool for case control studies

| **STUDY** | **ITEM 1** | **ITEM 2** | **ITEM 3** | **ITEM 4** | **ITEM 5** | **ITEM 6** | **ITEM 7** | **ITEM 8** | **ITEM 9** | **ITEM 10** | **INCLUDE** | **EXCLUDE** | **SEEK FURTHER INFO** | **SCORE (mean)** |
| --- | --- | --- | --- | --- | --- | --- | --- | --- | --- | --- | --- | --- | --- | --- |
| Carmignani et al., 2018 | Y | Y | Y | Y | Y | U | U | Y | Y | Y | Y |  |  | 100% |

*Legend:* Y=Yes; N=No; U=Unclear; NA=Not Applicable / Items from *JBI Critical appraisal tool for case control studies:* *1.Were the groups comparable other than the presence of disease in cases or the absence of disease in controls?; 2.Were cases and controls matched appropriately?; 3.Were the same criteria used for identification of cases and controls?; 4.Was exposure measured in a standard, valid and reliable way?; 5.Was exposure measured in the same way for cases and controls?; 6.Were confounding factors identified?; 7.Were strategies to deal with confounding factors stated?; 8.Were outcomes assessed in a standard, valid and reliable way for cases and controls?; 9.Was the exposure period of interest long enough to be meaningful?; 10.Was appropriate statistical analysis used?*

**Table S4.** Critical appraisal of cohort studies included

| **STUDY** | **ITEM 1** | **ITEM 2** | **ITEM 3** | **ITEM 4** | **ITEM 5** | **ITEM 6** | **ITEM 7** | **ITEM 8** | **ITEM 9** | **ITEM 10** | **ITEM 11** | **INCLUDE** | **SCORE (mean)** |
| --- | --- | --- | --- | --- | --- | --- | --- | --- | --- | --- | --- | --- | --- |
| Ahlberg et al.,  2011 | Y | Y | Y | Y | Y | Y | Y | Y | Y | Y | Y | Y | 100% |

Legend: Y=Yes; N=No; U=Unclear; NA=Not Applicable; / Items from JBI Critical appraisal checklist for Cohort Study: 1= Were the two groups similar and recruited from the same population? 2= Were the exposures measured similarly to assign people to both exposed and unexposed groups? 3= Was the exposure measured in a valid and reliable way? 4= Were confounding factors identified? 5= Were strategies to deal with confounding factors stated? 6= Were the groups/participants free of the outcome at the start of the study (or at the moment of exposure)? 7= Were the outcomes measured in a valid and reliable way? 8= Was the follow-up time reported and sufficient to be long enough for outcomes to occur? 9= Was follow-up complete, and if not, were the reasons to loss to follow up described and explored? 10= Were strategies to address incomplete follow-up utilized? 11= Was appropriate statistical analysis used?

**Table S5.** JBI. Critical appraisal tool of analytical cross-sectional studies

| **STUDY** | **ITEM 1** | **ITEM 2** | **ITEM 3** | **ITEM 4** | **ITEM 5** | **ITEM 6** | **ITEM 7** | **ITEM 8** | **INCLUDE** | **EXCLUDE** | **SEEK FURTHER INFO** | **SCORE (mean)** |
| --- | --- | --- | --- | --- | --- | --- | --- | --- | --- | --- | --- | --- |
| Pauli et al.,  2014 | Y | Y | Y | Y | Y | Y | Y | Y |  |  |  | 100% |

*Legend:* Y=Yes; N=No; U=Unclear; NA=Not Applicable / Items from Critical appraisal of analytical cross sectional studies: 1= Were the criteria for inclusion in the sample clearly defined?2=Were the study subjects and the setting described in detail?, 3=Was the exposure measured in a valid and reliable way?, 4=Were objective, standard criteria used for measurement of the condition?, 5=Were confounding factors identified?, 6=Were strategies to deal with confounding factors stated?, 7=Were the outcomes measured in a valid and reliable way?, 8=Was appropriate statistical analysis used?
